# Parameter Reliability and Understanding Enzyme Function

**DOI:** 10.3390/molecules27010263

**Published:** 2022-01-01

**Authors:** Andrew G. McDonald, Keith F. Tipton

**Affiliations:** School of Biochemistry and Immunology, Trinity Biomedical Sciences Institute, Trinity College Dublin, D02 PN40 Dublin, Ireland; ktipton@tcd.ie

**Keywords:** enzyme kinetics, Michaelis constant, maximal velocity, substrate competition, enzyme inhibition, cooperativity, bisubstrate kinetics, parameter uncertainties

## Abstract

Knowledge of the Michaelis–Menten parameters and their meaning in different circumstances is an essential prerequisite to understanding enzyme function and behaviour. The published literature contains an abundance of values reported for many enzymes. The problem concerns assessing the appropriateness and validity of such material for the purpose to which it is to be applied. This review considers the evaluation of such data with particular emphasis on the assessment of its fitness for purpose.

## 1. Introduction

Studies of the behaviour of an enzyme would not get far without knowledge of the Michaelis constant ($K_{m_{}}$) and maximum velocity ($V_{\max_{}}$) towards its substrates, since these are definitive features of enzyme activity. These are not strictly constants, but parameters, because they are dependent on conditions such as temperature, pH, and ionic strength. Many applications, including those listed below, rely on knowledge of enzyme kinetic parameters. At the simplest level, the $K_{m_{}}$ value, the substrate concentration which gives half maximum velocity, is an indication of the substrate concentration necessary for assaying an enzyme, whereas for deterministic systems modelling, which relies on experimentally measured parameters that can be represented by a series of linked ordinary differential equations (ODEs), these parameters are essential.

- Designing appropriate enzyme assay procedures
- Competition between different enzymes for a single substrate
- Competition between substrates for a single enzyme
- Understanding the effects of pH on enzymes
- Determination of enzyme-catalysed reaction mechanisms
- Assessment of inhibition and its effects
- Calculation of reaction equilibria under defined conditions
- The effects of temperature on enzyme activity
- Deterministic systems modelling (simulation)

**Figure 1:**
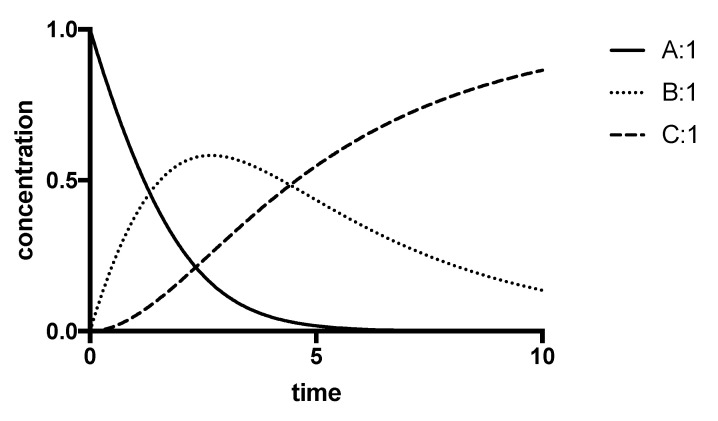
Behaviour of a simple two-enzyme model system. $V_{f}$ and $V_{b}$ are the maximum velocities $k_{\mathrm{cat}}\left[E\right]$) of E1 operating in the forward and backward directions, respectively. $K_{\mathrm{mf}}$ and $K_{\mathrm{mb}}$ are the respective Michaelis constants, and $V^{\prime }$ and $K_{m}^{\prime }$ are the maximum velocity and $K_{m_{}}$ value for E2. Square brackets indicate concentrations. Lower panel: time-course of the reaction with $V_{f}=1$, $V^{\prime }=2.5$, $K_{\mathrm{mf}}=1$, $K_{m}^{\prime }=10$ and $V_{b}=0$. Initial values: A = 1; B = 0; C = 0.

The steady-state kinetic relationships describing each of the above can be found in most biochemistry textbooks and many more specific books, with that by Segel [1] being exhaustive in its coverage, as well as in detailed articles (e.g., [2,3,4,5]). Their application is dependent on the values of the kinetic parameters used. Thus, it is important to understand the reliability of reported values or how to ensure the accuracy and precision of values that you determine yourself. The basic approach to deterministic systems modelling can be illustrated by the simple example of a sequence of reactions catalysed by two enzymes shown in Figure 1.

$$A\;\;\overset{E1}{\underset{}{⇌}}\;\;B\overset{E2}{⟶}C$$

(1) $$\begin{array}{ccc} \frac{d[A]}{dt} & = & -\frac{V_{\mathrm{mf}}\left[A\right]}{K_{\mathrm{mf}}+\left[A\right]}+\frac{V_{\mathrm{mb}}\left[B\right]}{K_{\mathrm{mb}}+\left[B\right]} \end{array}$$

(2) $$\begin{array}{ccc} \frac{d[B]}{dt} & = & \frac{V_{\mathrm{mf}}\left[A\right]}{K_{\mathrm{mf}}+\left[A\right]}-\left(\frac{V_{\mathrm{mb}}\left[B\right]}{K_{\mathrm{mb}}+\left[B\right]}+\frac{V^{\prime }\left[B\right]}{K_{m}^{\prime }+\left[B\right]}\right) \end{array}$$

(3) $$\begin{array}{ccc} \frac{d[C]}{dt} & = & \frac{V^{\prime }\left[B\right]}{K_{m}^{\prime }+\left[B\right]} \end{array}$$

Integration of such a relationship will give the time courses of the formation of each of the intermediate and steady-state concentrations of B when d[B]/d*t* = 0 and the rate of C formation is constant. The chain can be expanded to involve additional enzyme-catalysed reactions, branching and converging pathways, as well as uncatalysed and transport reactions. Each of the individual steps may be refined to take into account the involvement of two or more substrates, as well as other complexities, such as product and high-substrate inhibition. Cooperativity and allosteric effects can be addressed by use of a reversible model of the Hill equation [6], an extended Monod, Wyman, and Changeux allosteric equation [7], or an approximate version of these [8]. This will result in a chain of ODEs representing each transformation in a metabolic process (see [9], for example). The only requirement is knowledge of the kinetic parameters and behaviour of each enzyme and the transport process in the system and the rates of any non-catalysed reactions that might be involved.

This seems relatively straightforward, but unless the parameters used are accurate and reliable, it will otherwise be—as stated in the now quite old adage—a case of “garbage-in, garbage-out”. Therefore, it is necessary to consider how appropriate values might be obtained and whether they have sufficient levels of accuracy and precision for the use to which they are intended.

## 2. Sources of Parameters

Assuming those attempting to simulate metabolic processes are unable, or unwilling, to determine the kinetic parameters for each enzyme in a pathway themselves, the first problem is to find the appropriate kinetic parameters to use. This may involve trawling through the original literature, but the process can be shortened by use of the BRENDA Enzyme information database [10], which contains kinetic parameters for many enzymes, from different sources, with references to the papers from which they were obtained. SABIO-RK [11], is another useful source of enzyme kinetic data. A problem with much of the reported material is that it is not always easy to find information on how the data were obtained and its reliability. The more recently developed STRENDA (STandards for Reporting ENzymology DAta) database [12] seeks to address these problems by establishing the information that should be made available when reporting such data. These are now a requirement for publishing in an increasing number of journals, which should facilitate access to more reliable data.

An additional, seemingly trivial point is to ensure that it is the appropriate enzyme for which you are collecting data. Several enzymes have been known by alternative names, and it is important to ensure that the correct one has been identified. Quite similar names may also be used by different enzymes; for example, there are two carbamoyl-phosphate synthases which differ in the source of ammonia that is added to bicarbonate: EC 6.3.4.16, carbamoyl-phosphate synthase (ammonia) and EC 6.3.5.5, carbamoyl-phosphate synthase (glutamine-hydrolysing). These enzymes not only differ in reactants, but the first of these requires *N*-acetyl-l-glutamate as an allosteric activator. There are also a number of oxidoreductases that differ in the coenzyme used. The best way to ensure you are dealing with the correct enzyme is through use of the EC classification numbers. These can be found on the International Union of Biochemistry and Molecular Biology (IUBMB) ExplorEnz website, which is the definitive list of EC numbers and enzyme names [13]. Although there are several other versions of this enzyme list available, the IUBMB cannot guarantee their accuracy. The ExplorEnz site allows any enzyme to be found through the other names that have been used for it. Since the EC classification system is based on the catalysed reaction, isoenzymes that do not differ significantly in that respect will have the same EC number, and thus it may also be necessary to specify the specific isoenzyme being examined. It is, of course, essential to use data obtained from the same species and organelle as the pathway being considered.

Distinguishing between isoenzymes can represent a particular problem. This can be illustrated by horse-liver alcohol dehydrogenase, which has been studied extensively and with samples of the purified enzyme available commercially. It would, therefore, seem reasonable to assume that all samples of that enzyme are similar. However, some horse livers have been shown to contain significant amounts of a different isoenzyme (SS) with different substrate specificities and kinetic parameters with respect to ethanol and acetaldehyde [14,15]. Thus, it would be a mistake to assume that data from an organ from one animal would be typical of that of the organs of the same animals. Such situations can be even more complex in bacteria where errors in transcription and translation may be even more commonplace [16].

## 3. Selecting Appropriate Assay Conditions

A more difficult problem has been that many investigators who have been interested in the kinetic behaviour of specific enzymes have used assay conditions that are favourable to their studies without regard for the physiological conditions under which the enzyme may operate in the tissues [17]. For example, many studies on the behaviour of alcohol dehydrogenase have involved alkaline pH values (e.g., [18]), simply because the reaction equilibrium favours aldehyde oxidation at neutral pH values [19], which have generally been used for studies in the reverse direction. It is, of course, unhelpful in studies of reversible reactions for different pH values to have been used for the forward and reverse directions, which would, for example, make it impractical to determine the equilibrium constant from Haldane relationships [1,5]. In other cases, non-physiological substrates have been used because their conversion is easier to monitor, although it is often unclear whether use of such substrates affects the kinetic behaviour of the enzyme in question [20].

The assay temperature should also be considered since 30 °C is most commonly encountered, but some older studies have used 25 °C, or the ambiguous ‘room temperature’. As examples, complete kinetic studies of glyceraldehyde-3-phosphate dehydrogenase (EC 1.2.1.22) have been reported at pH 8.6 and 30 °C for the enzyme from rabbit muscle [21], and kinetic data for hexokinase from rat muscle have been reported at pH 6.0 [22], whereas rat liver glucokinase has been studied at pH 8.0 [23].

The variety of different buffer systems, chelating and non-chelating, and additives that have been used in assays is quite bewildering, and it has generally been believed that the only consideration is to use a system in which the enzyme is stable and the assay is practicable, regardless of the conditions in which the enzyme may operate in vivo. However, the system used may have more fundamental effects on the enzyme behaviour by affecting their conformational motions [24]. Furthermore, the buffer components used may have differential effects; for example, phosphate and potassium ions activate some enzymes, but inhibit others [25,26,27,28,29,30,31,32,33,34,35,36,37,38,39], and purified ‘native’ glutamate dehydrogenase is stable in phosphate buffer, but not in Tris buffer [28], whereas Tris and HEPES inhibit carbamoyl synthase (ammonia) [29].

Such considerations makes integration of the data into a single metabolic system a complex operation. Attempts have been made to concoct assay media that mimic the conditions that occur within the cell type under study [30], although cellular pH is known to vary with metabolic conditions [31,32].

## 4. Simple Questions That Have to Be Asked

Authors should discuss the results and how they can be interpreted from the perspective of previous studies and of the working hypotheses. The findings and their implications should be discussed in the broadest context possible. Future research directions may also be highlighted.

It ought not be necessary to ask whether reported studies were based on initial rate measurements, and one might expect authors to state that this was so. Determination of kinetic parameters according to the Michaelis-Menten equation is predicated on the initial rate being determined, since the rate may decrease over time owing to substrate depletion, product inhibition, enzyme instability, or some other cause. During the initial, linear, or rate period, none of these should have had time to operate. Many assay procedures involve determining the amount of product formed after a fixed time, and their use has increased with the development of high-throughput assays. Thus, it is important to be assured that initial rate values were obtained under all conditions used in a study.

Since enzymes are catalysts which are usually used at much lower concentrations than those of their substrates, it would be expected that the initial velocity would be proportional to the enzyme concentration. Clearly, if this were not the case, $V_{\max_{}}$ would have no useful meaning. There are a number of possible cases where such strict proportionality does not occur, including dissociation of a multimeric enzyme to a form with different activity, the presence of dissociable inhibitors or activators in enzyme preparation, or contamination of the substrate by small amounts of an irreversible inhibitor. Although such effects are, fortunately, rare, it should be incumbent on authors to indicate that proportionality has been established.

**Figure 2:**
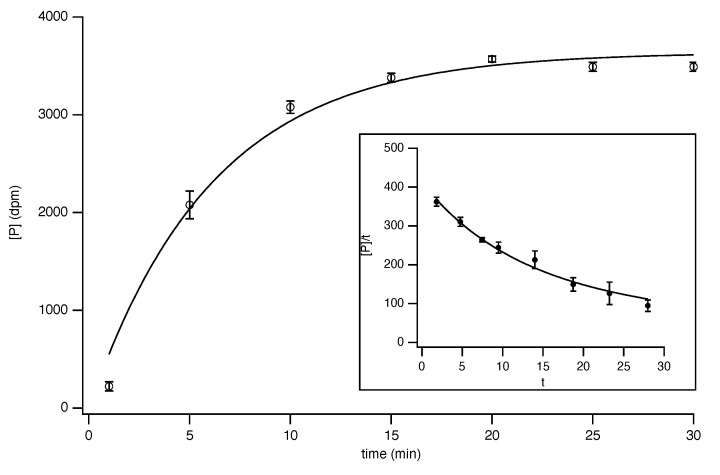
Time-course of the oxidation of 2-phenylethylamine by monoamine oxidase-B. Activity was determined by a radiochemical procedure. Each point is the mean ± s.e.m. from three determinations, with some points at longer times omitted. The inset shows a representative plot of [P]/time against time to determine the initial rate.

Sometimes, it is not possible to use a substrate concentration that is sufficiently high to avoid significant depletion during the course of the assay, and the progress curve appears to be a continuous curve, as shown, for example, in Figure 2. If substrate depletion is the sole cause of the fall-off, it is possible to treat such curves as first-order reactions and express the activity as a first-order rate constant [33]. Such a procedure has been used to determine the activity of peptidases, such as papain, which have quite high $K_{m_{}}$ values [34]. More elaborate curve-fitting procedures have been applied to allow statistical evaluation and increased accuracy (see [40,41]); however it should be recognised that, owing to mixing and start-time variability, the zero-time value may be inaccurate and it should not be assumed that the progress curve passes through the origin ([P] = 0 when *t* = 0). A more direct procedure, which makes no assumptions, is to measure the amount of product formed ([P]) at a series of times (*t*) and plot [P]/*t* against *t*, which will give a curve which will extrapolate to intersect the x axis at a value corresponding to the initial rate (Figure 2). The same result will be obtained if [P]/*t* is plotted against [P].

It is also necessary to check that the activity of the enzyme has been reported in some meaningful way. The most commonly used quantity is the Unit, sometimes referred to as the International Unit or Enzyme Unit. One Unit of enzyme activity is defined as that catalysing the conversion of 1 μmol substrate (or the formation of 1 μmol product) in 1 min. The specific activity of enzyme preparation is the number of Units per mg protein. Some workers, however, use the term unit to refer to more arbitrary measurements of enzyme activity, such as absorbance change per unit time per mg enzyme protein, so it is essential that the units used are defined in any publication. Since minutes and μmol are not recognised for SI (Système international) units, their official recommendation is the Katal (abbreviated to kat), one Katal corresponding to the conversion of 1 mol of substrate per second. This it is an inconveniently large quantity compared to the Unit. The relationships between Katals and Units are

$$\begin{array}{ccc} 1\;\mathrm{kat} & = & 60\;\mathrm{mol}\cdot \min^{-1}=6\;\times 10^{7}\;\mathrm{Units}\\ 1\;\mathrm{Unit} & = & 1\;\mu \mathrm{mol}\cdot \min^{-1}=16.67\;\mathrm{nkat}. \end{array}$$

If the molar enzyme concentration (e) is known, the activity is sometimes expressed as the rate constant $k_{\mathrm{cat}}=V_{\max_{}}/e$, with the dimension of time${}^{-1}$, usually s${}^{-1}$. This may be less than helpful if the cellular enzyme concentration is unknown. Even here, some caution may be necessary, since some workers have treated oligomeric enzymes in terms of the number of catalytic subunits rather than as a single entity. This gives catalytic centre activity, which corresponds to the mol substrate used, or product formed, per min per catalytic centre (active site). Thus, for a tetrameric protein, there might be a 4-fold difference between units per mol, and units per subunit. Such calculations ignore the possibility of ‘half-site reactivity’ where the active sites of a multimeric enzyme operate alternately, which means that one-half of the identical subunits is active at any one time [35]. Since this phenomenon has been demonstrated, or inferred, for a number of oligomeric enzymes, catalytic centre activities should be viewed with caution. There is one more simple question to be considered—that of the reaction stoichiometry and which product or substrate was monitored. For example, for carbamoyl-phosphate synthetase (ammonia) (EC 6.4.3.16) catalyses:

$$2\;\mathrm{ATP}+{\mathrm{NH}}_{3}+\mathrm{hydrogencarbonate}=2\;\mathrm{ADP}+\mathrm{phosphate}+\mathrm{carbamoylphosphate}$$

Therefore, the activity determined by measuring ADP production will be twice that observed if carbamoyl phosphate formation is measured.

These points indicate that there may be a need to peruse the original literature carefully before simply accepting the published $K_{m_{}}$ and $V_{\max_{}}$ values uncritically.

## 5. What Effective Parameters Might Be Needed

The simple schemes shown in Figure 1 involved enzymes catalysing reactions involving a single substrate, whereas most enzymes catalyse reactions involving two or more substrates. The steady-state kinetics of such enzyme-catalysed reactions have been described in many books and articles, including those referred to above [1,2,3,4,5]. The reaction of an enzyme involving the conversion of two substrates can be represented as that of a donor–acceptor pair:

$$\mathrm{Ax}+B\;\overset{}{\underset{}{⇌}}\;A+\mathrm{Bx}.$$

The general kinetic equation for such a reaction can be written as:

(4) $$\begin{array}{ccc} v & = & \frac{V_{\max_{}}\left[\mathrm{Ax}\right]\left[B\right]}{\left[\mathrm{Ax}\right]\left[B\right]+K_{m_{\mathrm{Ax}}}\left[B\right]+K_{m_{B}}\left[\mathrm{Ax}\right]+K_{s_{\mathrm{Ax}}}K_{m_{B}}} \end{array}$$

(5) $$\begin{array}{ccc} & = & \frac{V_{\max_{}}}{1+\frac{K_{m_{\mathrm{Ax}}}}{\left[\mathrm{Ax}\right]}+\frac{K_{m_{B}}}{\left[B\right]}+\frac{K_{s_{\mathrm{Ax}}}K_{m_{B}}}{\left[\mathrm{Ax}][B\right]}}, \end{array}$$

where the parameter $K_{s_{\mathrm{Ax}}}$ (sometimes represented as $K_{i_{\mathrm{Ax}}}$) is the apparent dissociation constant for the first substrate to bind, as the concentration of the second substrate falls to zero. This lower equation is written in a form that has the advantage that all terms in the denominator are dimensionless [36] (see also [5]).

This general equation applies to sequential reactions in which one substrate must bind before the other and random-order mechanisms under quasi-equilibrium conditions, where $K_{s_{\mathrm{Ax}}}K_{m_{B}}=K_{m_{\mathrm{Ax}}}K_{s_{B}}$. The term $K_{s_{\mathrm{Ax}}}K_{m_{B}}$ is absent from the equation for enzymes following a double-displacement (ping-pong) mechanism except when products are present, when $K_{s_{\mathrm{Ax}}}$ is more complex [5,36].

The initial rate equation shows that the apparent $K_{m_{}}$ values for each substrate will be dependent on the concentration and Michaelis constant(s) of the other.

$$K_{m_{\mathrm{Ax}}}^{\mathrm{app}}=\frac{1+\frac{K_{m_{B}}}{\left[B\right]}}{K_{m_{\mathrm{Ax}}}+\frac{K_{s_{\mathrm{Ax}}}K_{m_{B}}}{\left[B\right]}};\;\;K_{m_{B}}^{\mathrm{app}}=\frac{1+\frac{K_{m_{\mathrm{Ax}}}}{\left[\mathrm{Ax}\right]}}{K_{m_{B}}+\frac{K_{s_{\mathrm{Ax}}}K_{m_{B}}}{\left[\mathrm{Ax}\right]}}$$

The true $K_{m_{}}$ value, the substrate that gives half maximum velocity, will be obtained only when the concentration of the other substrate is saturating ([S] ≫$K_{m_{}}$).

It follows that it is necessary to know the $K_{m_{}}$ values of each substrate and the concentration of the second substrate, as well as which substrate binds to the enzyme first in an ordered mechanism. Therefore, rather than attempting to adjust the parameters for the simple single-substrate Michaelis equation, the full kinetic equation for each enzyme, including product inhibition constants, if they are significant, should be used. Although these may appear somewhat unwieldy, the terms containing $K_{m_{B}}/\left[B\right]$ can be treated as constants if the steady-state concentration of B is known. Fortunately, many reactions involve a coenzyme, such as NAD(P)${}^{+}$, ATP, or more commonly, its complex with Mg${}^{2+}$ ions, acetyl-CoA, and so forth, and the steady-state concentrations of these may be available from the literature. Of course, several enzyme catalysed reactions involve more than two substrates, requiring a knowledge of more $K_{m_{}}$ values and steady-state co-substrate concentrations. For example, the rate equation for carbamoyl phosphate synthase (EC 2.7.2.5), which involves four substrates, contains 24 separate terms in the denominator [37]. This would lead to unwieldy, but nevertheless tractable, equations to consider.

The ratio $k_{\mathrm{cat}}/K_{m_{}}$ has been termed the specificity constant [38], since it represents the apparent first-order rate constant for a single substrate reaction when [S] $\ll K_{m_{}}$. When two substrates, A and B, compete for a single enzyme,

$$\frac{v_{a}}{v_{b}}=\frac{V_{a}\left[A\right]}{K_{m_{a}}}\frac{K_{m_{b}}}{V_{b}\left[B\right]}=\frac{\left(k_{{\mathrm{cat}}_{a}}/K_{m_{a}}\right)}{\left(k_{{\mathrm{cat}}_{b}}/K_{m_{b}}\right)}\frac{\left[A\right]}{\left[B\right]}.$$

For a reaction involving two substrates, this will depend on the kinetic mechanism. For a compulsory-order mechanism, it will only be the case at saturating concentrations of the second substrate. Under real conditions, for such a two-substrate reaction, the effective value of $k_{\mathrm{cat}}$ ($k_{\mathrm{cat}}^{\prime }$) will be dependent on the concentration of the second substrate.

$$k_{\mathrm{cat}}^{\prime }=k_{\mathrm{cat}}/\left(1+K_{m_{b}}/\left[B\right]\right)$$

Since for two substrates competing for a single enzyme, one can assume that [B] will be the same for both substrates, but it would be necessary to take account of these factors, including the kinetic mechanism operative, before trying to calculate specificity constants from values reported in the literature. It should also be noted that the use of specificity constants to compare the activities of two enzymes acting on a single substrate is invalid [39], since, as discussed below, different relationships apply in such cases [5,42].

## 6. Pitfalls of Using Data from Purified Enzymes

Most detailed kinetic studies have used purified enzyme preparations, since that minimizes the possibility of interfering reactions in assay and purification is generally a pre-requisite for establishing the reaction catalysed. However, there may be problems with that approach, since some enzymes are regulated by covalent modification, such as phosphorylation, acylation, or adenylation, and these may be lost on purification. For example, in the purification of glycogen phosphorylase, it is necessary to ensure that the contaminating kinases and phosphorylases are removed or inactivated [43]. Furthermore, the purification process itself may modify the enzyme, for example, a standard purification procedure that had been used for the enzyme fructose-bisphosphatase (EC 3.1.3.11) resulted in limited proteolysis that altered its pH optimum and decreased its sensitivity to allosteric inhibition by AMP [44]. Unfortunately, it appears that data from such artefactually modified systems were used in some earlier simulation studies [45]. Limited proteolysis of glutamate dehydrogenase [NAD(P)${}^{+}$] (EC 1.4.1.3), which can occur during purification, alters its kinetic and inhibitory properties [46]. Furthermore, the purified native enzyme is sensitive to atmospheric oxidation of an N-terminal cysteine residue, resulting in changes to its inhibitor sensitivity [47]. Some other enzymes require an additional component for their function, for example, the enzyme *N*-acetyllactosamine synthase (EC 2.4.1.90) is converted to lactose synthase (EC 2.4.1.22) by binding α-lactalbumin [48], and a number of multi-subunit enzymes require non-catalytically active proteins for their assembly and activity regulation (e.g., [49,50,51,52,53]), whereas non-catalytic components are necessary for some substrates to access their metabolising enzymes within organelles [52].

Glycosylation proved troublesome for many studies that used cloned and expressed enzymes, since some earlier studies used systems that were deficient in this respect. It was claimed that glycosylation was of little importance, despite the fact that with some enzymes, it presented a hydrophilic layer through which the substrate had to penetrate to reach the active site. It has been shown that glycosylation is important for correct enzyme secretion and localization, but it is also important for the activity of some enzymes [54,55].

Such considerations indicate the importance of ensuring that the enzymes for which kinetic studies are obtained represent the enzyme, as it occurs in vivo and not as an incomplete or modified protein, if interpretations of their behaviour within the tissues are to have any validity.

## 7. Approximating the Activity within the Cell

Although it may be possible to estimate the activities of some enzymes in vivo [56,57], this has, so far, been restricted to only a few, and is of course limited to extra-cellular enzymes and those with substrates that can readily penetrate cells [58]. When full purification data for an enzyme have been reported, it may be possible to work backwards from the activity in crude extracts to that within the intact cell; however, that is not possible for cloned preparations, and the citation of $k_{\mathrm{cat}}$ values rather than maximum velocities adds further uncertainties, requiring a measure of the enzyme protein concentration. There are data for the protein abundance in some cell types [59,60], but initial hopes that it might be possible to determine expressed protein concentrations from determining mRNA levels have proved invalid [61]. With some enzymes, it may be possible to estimate the amount present in disrupted cells from antibody-binding measurements [62], or from the binding-specific irreversible inhibitors [63]. However, the amount of any specific enzyme protein present does not necessarily correspond with its activity. The rates of enzyme synthesis and degradation vary widely, and the amount of an enzyme within the cell is not constant and can vary as a result in changes in metabolic conditions [64,65].

**Figure 3:**
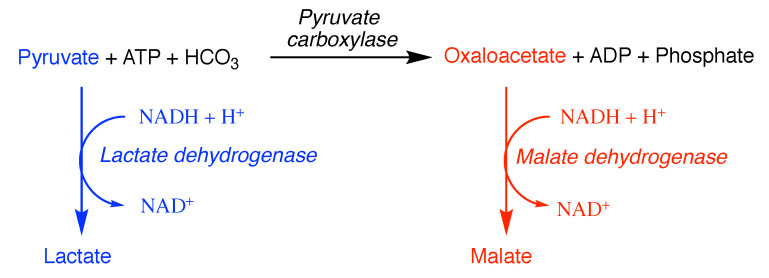
Interference with the assay of pyruvate carboxylase. The assay, based on measurement of malate dehydrogenase-catalysed reduction of oxaloacetate oxidation by NADH + H${}^{+}$, which can be followed spectrophotometrically at 340 nm, is shown in red, with the interfering reaction catalysed by lactate dehydrogenase in blue.

Therefore, one is driven to measuring the activities in disrupted cells or isolated organelles and using the $V_{\max_{}}$ values to estimate the in vivo activities [30,66,67]. It is often not possible to determine classical initial-rate kinetics for many enzymes in vivo, since that would necessitate conditions where the basal substrate concentrations, as well as those of any effectors were zero. Despite the caveats, discussed above, the accepted way is to measure the enzyme activity in disrupted cells to determine the maximum velocity, and then rely on a detailed kinetic analysis performed separately with purer preparations of the enzyme. It should be noted that some standard assay procedures will not function in the presence of other contaminating enzymes [68]; for example, a standard assay for pyruvate carboxylase (EC 6.4.1.1) involves the addition of malate dehydrogenase to couple the oxaloacetate produced to the oxidation of NADH, which may be followed spectrophotometrically (Figure 3). If the enzyme preparation is contaminated with lactate dehydrogenase, this will also catalyse the oxidation of NADH in converting pyruvate to lactate. Clearly, in this case, it is not possible to exclude pyruvate from the assay mixture, since it is a substrate for the enzyme being assayed. An alternative assay, such as the determination of the incorporation of radioactively labelled bicarbonate into oxaloacetate, must be used with impure preparations of the enzyme because the interference from lactate dehydrogenase might not be expected to be important in the absence of added NADH [69]. The coupled assay can only be used satisfactorily if the pyruvate carboxylase preparation is purified to a state where it is free from contaminating lactate dehydrogenase.

A further, not completely resolved, problem is that of crowding [70]. Most kinetic studies have involved isolated enzymes in simple buffer systems, whereas the situation in the cell or organelle is considerably more complex, with many different protein and other molecules resulting in an overcrowded environment. Whereas the kinetics of enzyme-substrate interactions has often assumed simple diffusion limited, or controlled, collision theory [71], the maximum value of $k_{\mathrm{cat}}/K_{m_{}}$ would be around $10^{8}$–$10^{9}$ M${}^{-1}$ s${}^{-1}$ if every collision between the enzyme and substrate molecule results in the formation of a viable ES complex. Not surprisingly, few enzymes approach this theoretical maximum value [72], but a few do so, and some even appear to exceed it (e.g., [73]). As might be expected, cellular crowding affects diffusion rates, as well as protein aggregation and phase separation [74]. Many attempts to quantify these effects have used ‘inert’ crowding additives, such as dextran, ficol, or polythylene glycol [75,76]. Although such studies indicated the kinetic behaviour to be selectively affected, the nature of the crowding agent itself was found to be an important factor [74,77]. Bovine serum albumin has been used in some studies as a more physiological crowding agent [78] and, perhaps, not surprisingly, the effects of this protein have been found to differ from those of dextran [78,79]. However, its use as a crowding agent requires caution since it binds many normal cellular components, including metal cations, some amino acids and peptides, ascorbate, steroids, and fatty acids [80]. Indeed, it is unlikely that a single crowding agent could mimic the situation in the cell, where there is a range of components differing in their surface-charge distributions and potential hydrophilic and hydrophobic interaction sites. In such a crowded environment, enzymes might be expected to bind non-specifically to other cellular components [44], which may also affect their behaviour, whereas the substrate may also bind to other cellular components, which, if reversible, would effectively buffer the free concentration at a lower value.

The multiple ionizable groups of macromolecules as well as small molecules all contribute to the ionic strength of the intracellular milieu, but this multiplicity of contributory factors makes any calculation of this value problematic. Sensor methods have been developed [81] and used to monitor ionic-strength changes in human kidney cells [82], but the possible effects of ionic strength on metabolism have been often ignored in kinetic and simulation studies. Although there have been some studies that indicate the effects of ionic strength on specific enzymes [83,84,85] and how it may affect intracellular pressure, protein folding, and aggregation [86,87], it has proved difficult to disentangle ionic-strength effects from those of the specific ionic species involved [26,86]. Ideally, any assay medium used for in vitro studies should mimic the cellular ionic strength, but that aspect has often been ignored.

Thus, although buffer systems that attempt to reproduce the ionic compositions and pH values of different cell types represent an improvement over unitary buffers [30], they fall short of mimicking the complexity of the environment in which the metabolism operates within the cell, and cannot be regarded as being constant.

## 8. Obtaining Reliable Data

### 8.1. Curve Fitting

Various methods have been used for determining enzyme kinetic parameters. Equations for linearizing the manipulation of the Michaelis-Menten equation to provide linear plots were first produced by Woolf, but subsequently, were often attributed to others [88]. These involve plots of 1/v versus 1/[S], [S]/*v* versus [S] or *v* versus *v*/[S] [5,36]. Since these all involve reciprocal values, the distributions of experimental points and error estimates will be distorted. Much earlier data were determined by the use of double-reciprocal (Lineweaver-Burk) plots of the initial-rate values, despite this being well-known to be the least accurate of the graphical procedures available. Dowd and Riggs [89] concluded that: “The undeserved popularity of the Lineweaver-Burk method may well be based upon just this ability to provide what seems to be a good fit even when the experimental data are poor”. However, the behaviour of reversible inhibitors are well-understood in terms of these plots, making their use valuable for illustrative purposes preferable, but parameters determined from such plots should be treated with some caution.

An alternative procedure for determining $K_{m_{}}$ and $V_{\max_{}}$ was introduced by De Miguel Merino [90] and by Eisenthal and Cornish Bowden [91]. This involves drawing a line connecting the substrate concentration on the horizontal axis with the corresponding velocity on the vertical axis. These lines for a number of different substrate concentrations will intersect at a point corresponding to $K_{m_{}}$ on the horizontal axis and $V_{\max_{}}$ on the vertical axis. The only difference between the two approaches is that negative substrate concentrations were used by Eisenthal and Cornish Bowden, with the intersection point corresponding to $K_{m_{}}$, whereas positive substrate concentrations were used by De Miguel Merino, with the intersection point corresponding to $-K_{m_{}}$. Normal experimental errors will result in there being a range of intersection points, and these can be used to give a non-parametric value of the confidence limits [92]. This procedure, which has been called the direct-linear plot, has been shown to be superior to the linearization plots. Although the type of reversible inhibition should be obvious from whether $V_{\max_{}}$ or $K_{m_{}}$, or both, are affected, it can be less clear if experimental error results in ranges of intersection points.

Despite Wilkinson having shown, as early as 1961 [93], that the least-squares method could be used to fit Michaelis curves directly to yield the kinetic parameters and their associated errors, the adoption of this procedure was relatively slow, until computers in laboratories became sufficiently widespread and appropriate programs were developed [94]. This is now recognized as the most accurate and robust procedure to use [95]. The accuracy of this approach requires more experimental points than are theoretically necessary for a straight line. The display of residuals is a good way of checking how well a fitted curve corresponds to the data, and most curve-fitting programs have this option. The residual for any point is defined as Δv=(observedv−calculatedv). When these are plotted against substrate concentration, they will show deviations from a straight line that are parallel to the substrate concentration axis. With experimental errors, the residuals might be expected to be evenly distributed above and below the line, but residuals that show a systematic deviation would indicate that the fit may not be appropriate [96].

### 8.2. The Importance of Zero

If one was to produce a simple calibration curve of signal versus concentration, it is important to check that it goes through the origin, with a zero signal at zero concentration. None of the linear solutions to the Michaelis-Menten equation allow the value of *v* when [S] = 0 is to be included in the graph. Therefore, blank rates that should have been subtracted from *v* values will not be apparent. This represents a further reason for fitting data to a Michaelis curve and ensuring that it does pass through the origin. Selwyn [97] has pointed out that if the true zero is not taken into account data, fitting a Michaelis curve will also give acceptable fits to an exponential curve and the equation describing a segment of a circle. If a blank rate is constant, failure to correct for it will result in a Michaelis-Menten curve that does not pass through the origin; finite activity at [S] = 0. A particular case is where a blank rate is proportional to substrate concentration. The situation is analogous to the non-specific binding that is common in studies of ligand-binding to receptors. It can be represented by the equation

$$v=\frac{V_{\max_{}}\left[S\right]}{K_{m_{}}+\left[S\right]}+k\left[S\right],$$

where *k* is the rate constant for the non-enzymic, blank rate. This will result in a curve of *v* versus [S] that tends to a constant rate (k[S]) rather than saturation as the substrate concentration is increased. In terms of a double-reciprocal plot, a curve that passes through the origin (*v* = *∞*, when [S] = *∞*) would result.

### 8.3. Progress-Curve Analysis

A different approach to the determination of kinetic parameters is to follow the complete time-course of the enzyme-catalysed reaction as the substrate becomes exhausted. This should have the theoretical advantage of using the entire progress curve rather than the small, initial-rate portion of it. Kinetic parameters can be calculated from an integrated rate equations describing the decline of activity as the substrate concentration falls or by direct curve-fitting procedures [98]. However, depletion of substrate may not be the only reason that the rate falls with time [36,67], so careful controls are necessary. Many computer applications have the facility to fit progress curve data or their integrated forms to allow the Michaelis constants to be obtained. The Lambert ω function, for example, has been shown to be an accurate method for determining Michaelis constants from progress curve data [99], using a closed-form solution to the Michaelis-Menten equation derived by Schnell and Mendoza [100]. Different users may have different preferences, and it would be of little value to make specific recommendations, but caution is necessary, since not all are free from errors [101].

## 9. Apparent Cooperativity

Some enzyme-catalysed reactions do not follow simple hyperbolic kinetics. High-substrate inhibition is usually easy to discern by a decline in activity at higher substrate concentrations, whereas apparently sigmoid curves may be explained by cooperative behaviour, best described by the Hill equation

(6) $$v=\frac{V_{\max_{}}{\left[S\right]}^{h}}{K+{\left[S\right]}^{h}},$$

where *h*, the Hill constant, is simply the power to which the substrate concentration should be raised to give normal saturation kinetics. Although the linear version of this equation has been widely used for calculating the values of *K* and *h*,

$$\log(v/\left(V_{\max_{}}-v\right)=\left(h\log\left[S\right]-\log K\right),$$

it is an approximation and is only linear over a limited range of substrate concentrations and direct curve-fitting procedures are more robust [102]. It should be noted that the constant *K* in the Hill equation does not represent the substrate concentration that gives half-maximum velocity (${\left[S\right]}_{0.5}$), but rather that value raised to the *h*th power, ${\left[S\right]}_{0.5}=\sqrt[{h}]{K}$.

Cooperativity, which is normally considered to be the binding of substrate to one subunit of an oligomeric protein affecting the affinity at other site(s) [103] is, however, not the sole cause of sigmoid curves and other possibilities should be considered before assuming the mechanism involved. Random order of substrate-binding, leading to a ternary complex that then breaks down to release products under steady-state conditions which will give a rate equation of the form:

$$v=\frac{(C_{1}ab+C_{2}a^{2}b+C_{3}ab^{2})\cdot e}{C_{4}+C_{5}a+C_{6}b+C_{7}ab+C_{8}a^{2}+C_{9}b^{2}+C_{10}a^{2}b+C_{11}ab^{2}},$$

where $C_{1}$–$C_{11}$ are each complex combinations of rate constants, *a* and *b* are the substrate concentrations and *e* is the enzyme concentration. This equation, which is the ratio of two polynomials, can give a variety of different curve shapes, including those resembling cooperative behaviour, depending on the relative values of the *C* constants and the substrate concentrations [104]. Only when the breakdown of the ternary complex is sufficiently slow for the substrate-binding steps to be in thermodynamic equilibrium will Michaelis-Menten behaviour be observed, for the other possible steady-state mechanisms involving activators and inhibitors can also, theoretically, give rise to complex kinetic behaviour [105]. However, true cooperativity is a ligand-binding phenomenon, and none of these mechanisms would be expected to give complex behaviour if the binding of substrates was measured at equilibrium.

Apparently, complex behaviour can also be the result of ill-considered experiments. A common example concerns the situation where the true substrate for its enzyme is its complex with a metal ion, such as Mg${}^{2+}$. If the substrate and metal ion are mixed together and varied in a fixed ratio, the concentration of the complexed species will vary with the amount of mixture added, resulting in an apparent sigmoid relationship between a velocity and substrate mixture concentration. Only if the concentration of Mg${}^{2+}$ is much greater that of the substrate and the complex dissociation constant can the mixture concentration be that of the complexed species. Otherwise, it will be necessary to calculate the concentration of the complex at each mixture concentration [106,107]. The situation can be more complicated if the true substrate is an activator-substrate complex, but the activator also binds to the free enzyme, as in the case of carbamoyl synthase (ammonia) (EC 6.3.4.16), where Mg${}^{2+}$ binds to the enzyme as an activator and the Mg-ATP complex is one of the substrates [108].

Negative cooperativity, where the value of the Hill constant (*h*) is less than 1, may be more difficult to see, as the Michaelis curve will be slower than normal to reach saturation; a doubly sigmoid curve will be obtained when *v* or saturation is plotted against the logarithm of substrate concentration, that is, where a fraction of the site(s) exhibits one dissociation constant and the rest exhibit(s) another. The condition for this to occur is that the ligand should also combine at another site on the enzyme, resulting in a decrease in affinity. Such behaviour has often been attributed to the binding of substrate to one site on an oligomeric enzyme causing a conformational change that decreases the affinity for other site(s) [103,109]. However, such behaviour does not necessarily involve more than one active site, and all that is required is that binding elsewhere on the enzyme reduces the affinity at the active site [110]. This essentially involves two enzyme species, where one is the free enzyme and the other is the enzyme with the alternative site occupied, acting on the same substrate. Indeed, such behaviour has, for example, been observed with trypsin (EC 3.4.21.4) [111]. Similar behaviour can occur if the same reaction is catalysed by two different enzymes that have different $K_{m_{}}$ values, as shown in the following reaction scheme, 
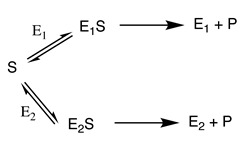
 where the rate of the overall reaction ($v_{\mathrm{total}}$) will be given by the sum of the velocities of the reactions catalysed by E${}_{1}$ and E${}_{2}$ ($v_{1}$ and $v_{2}$, respectively)

$$v=v_{1}+v_{2}=\frac{V_{\max_{1}}}{1+\frac{K_{m_{1}}}{\left[S\right]}}+\frac{V_{\max_{2}}}{1+\frac{K_{m_{2}}}{\left[S\right]}},$$

where $K_{m_{1}}$ and $K_{m_{2}}$ are the $K_{m_{}}$ values for E${}_{1}$ and E${}_{2}$, respectively, $V_{1}=k_{\mathrm{cat}}$ [E${}_{1}$] and $V_{2}=k_{\mathrm{cat}}^{\prime }$ [E${}_{2}$].

This equation does not describe a rectangular hyperbola for the velocity–substrate concentration curve. In fact, the form of the curve obtained [35] may be quite similar to that given by negative cooperativity. A procedure based on linearized plots (s/v versus *s*) to determine the kinetic parameters for each of the two enzymes has been reported [112]. Curve-fitting the data to the Hill equation with a value of *h* less than unity or the two enzyme model would both give respectable results. Hence, it is important to ensure that one is dealing with a single, homogeneous enzyme before concluding that negative cooperativity is involved.

## 10. Statistical Considerations

Whatever procedure is used to determine the parameters, it should also be noted that if, for example, a $K_{m_{}}$ value is given as x+y (n=5), that is unlikely to represent what a statistician might expect to be the mean ± SEM of five independent determinations of $K_{m_{}}$, but simply the curve-fitting error from points on a linear plot or a fitted Michaelis-Menten curve with five experimental points. The increased use of high-throughput micro-titre plate-based assays can also result in misleading statistical values. For example, a value cited as x+y (n=6) may simply mean that there were six of the values determined in the same set of plates and that the error only reflects pipetting errors, which would be expected to be small if automatic multi-pipettes were used [113]. Thus, one should be cautious of statistics that look better but actually mean less. A detailed treatment of statistics is beyond the scope of the present account, but some insightful comments on the meaning and interpretation of statistics have been published by Vaux [114,115]. However, that may well leave a significant number of reported kinetic parameters for the enzyme in which you are interested where none of the reasons discussed above would justify their exclusion. That raises the question of how they should be averaged.

### Distributions of Parameters

**Figure 4:**
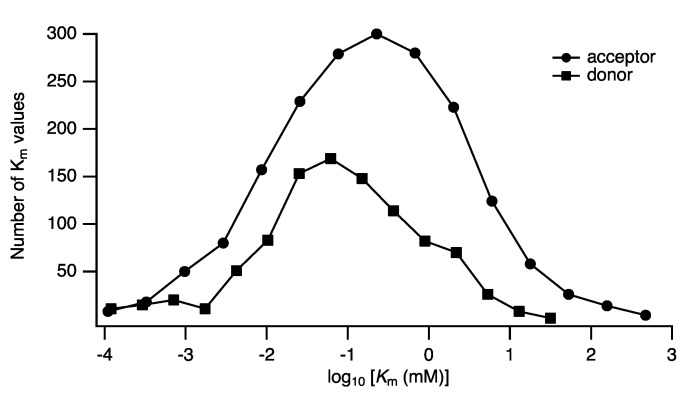
Distribution of $K_{m_{}}$ values among the glycosyltransferases. The acceptor and donor substrates of the hexosyltransferases and sialyltransferases are seen to have distinct $K_{m_{}}$ probability distributions, geometric means and geometric standard deviations (see text for details). Data are adapted from the BRENDA database [10].

In the statistical treatment of experimental errors, it is assumed that they are samples drawn from a population with a Gaussian arithmetic mean. For the parameters’ values themselves, however, given the nature of catalysis, we expect the distribution of parameters to be geometric, since their apparent values vary geometrically with temperature and pH. It is appropriate, therefore, to calculate means and standard deviations of the parameter values within logarithmic space. We illustrate with examples derived from data available in the BRENDA database [10]. The distribution of $K_{m_{}}$ values in each EC class is distributed geometrically (https://www.brenda-enzymes.info/functional_parameter_statistics.php (accessed on 6 December 2021)). A specific example is that of the glycosyltransferases, which transfer monosaccharides from a nucleotide-sugar donor to an acceptor. The acceptor varies widely, and can be a simple sugar, an oligosaccharide, protein, lipid, or polynucleotide. The reported $K_{m_{}}$ values of all donor and acceptors in the hexosyltransferase (EC 2.4.1.-) and sialyltransferase (EC 2.4.99.-) sub-subclasses were extracted from BRENDA. The distributions of the $K_{m_{}}$ values is shown in Figure 4. Since the original data have a heavily skewed distribution, an arithmetic mean and standard deviation are not meaningful, and a geometric mean (GM), $\bar{x}=e^{\frac{1}{n}\sum \log x_{i}}$, and the geometric standard deviation (GSD), s(x), given as

$$s\left(x\right)=\exp{\left(\frac{\sum {\left(\ln\frac{x_{i}}{\bar{x}}\right)}^{2}}{n}\right)}^{\frac{1}{2}}$$

is more appropriate. The value s(x) expresses a factor of the mean instead of a difference, thus approximately 68% of the values are expected to lie in the interval {GM/GSD, GM × GSD}. In the example given in Figure 4, the expected $K_{m_{}}$ values of glycosyltransferase acceptors (n=1850) were found to be distinct from those of the nucleotide-sugar donors (n=962), with calculated geometric means of 0.33 mM (GSD: 13.0) and 0.15 mM (GSD: 8.7), respectively. Such meta-studies, using data derived from BRENDA or SABIO-RK, could be useful in determining appropriate parameter ranges for use in systems-biological models.

## 11. Conclusions

Data-mining of enzyme kinetic data from the original literature is an unnecessarily complicated business that necessitates careful reading of the literature to ensure the values are appropriate to the proposed use. Further development of the STRENDA initiative [116] should help to ensure the relevance and trustworthiness of newly reported data, but significant quantities of potentially valuable legacy data remain, and here there is no alternative to careful evaluation to determine its accuracy and relevance. Perhaps the ideal answer would be to determine the parameters personally, under the conditions appropriate for the intended application and with suitably robust analytical methods. However, that may be too big of an ask for some, and it is hoped that the considerations described above may help to separate the useful from the less-appropriate data obtained from the literature.

## References

[1] Segel I.H. (1975). Enzyme Kinetics: Behaviour and Analysis of Rapid Equilibrium and Steady-State Enzyme Systems.

[2] Cleland W.W. (1967). Enzyme Kinetics. Annu. Rev. Biochem..

[3] Dalziel K. (1969). The interpretation of kinetic data for enzyme-catalysed reactions involving three substrates. Biochem. J..

[4] Fromm H.J. (1979). Summary of kinetic reaction mechanisms. Methods Enzymology.

[5] McDonald A.G., Tipton K.F., Horváth I.T. (2002). Kinetics of Catalyzed Reactions—Biological. Encyclopedia of Catalysis.

[6] Hofmeyr J.H.S., Cornish-Bowden H. (1997). The reversible Hill equation: How to incorporate cooperative enzymes into metabolic models. Bioinformatics.

[7] Najdi T.S., Yang C.R., Shapiro B.E., Hatfield G.W., Mjolsness E.D. (2006). Application of a generalized MWC model for the mathematical simulation of metabolic pathways regulated by allosteric enzymes. J. Bioinform. Comput. Biol..

[8] Heijnen J.J. (2005). Approximative kinetic formats used in metabolic network modeling. Biotechnol. Bioeng..

[9] Rizzi M., Baltes M., Theobald U., Reuss M. (1997). In vivo analysis of metabolic dynamics in *Saccharomyces cerevisiae*: II. Mathematical model. Biotechnol. Bioeng..

[10] Chang A., Jeske L., Ulbrich S., Hofmann J., Koblitz J., Schomburg I., Neumann-Schaal M., Jahn D., Schomburg D. (2021). BRENDA, the ELIXIR core data resource in 2021: New developments and updates. Nucleic Acids Res..

[11] Wittig U., Rey M., Weidemann A., Kania R., Müller W. (2018). SABIO-RK: An updated resource for manually curated biochemical reaction kinetics. Nucleic Acids Res..

[12] Swainston N., Baici A., Bakker B.M., Cornish-Bowden A., Fitzpatrick P.F., Halling P., Leyh T.S., O’Donovan C., Raushel F.M., Reschel U. (2018). STRENDA DB: Enabling the validation and sharing of enzyme kinetics data. FEBS J..

[13] McDonald A.G., Boyce S., Tipton K.F. (2009). ExplorEnz: The primary source of the IUBMB enzyme list. Nucleic Acids Res..

[14] Pietruszko R. (1974). Polymorphism of horse liver alcohol dehydrogenase. Biochem. Biophys. Res. Commun..

[15] Pietruszko R., Ryzewski C.N. (1976). A new subunit of horse liver alcohol dehydrogenase and subunit composition of the polymorphic form. Biochem. J..

[16] Ninio J. (1991). Connections between translation, transcription and replication error-rates. Biochimie.

[17] Boyce S., Tipton K.F., McDonald A.G., Hicks M.G., Kettner C. (2004). Extending enzyme classification with metabolic and kinetic data: Some difficulties to be resolved. Proceedings of the 1st International Beilstein Workshop on Experimental Standard Conditions of Enzyme Characterizations.

[18] Markovicč O., Theorell H., Rao S., Omfeldt M., Lagerlund I., Ehrenberg L. (1971). Rat Liver Alcohol Dehydrogenase. Purification and Properties. Acta Chem. Scand..

[19] McDonald A.G., Tipton K.F., Boyce S. (2009). Tracing metabolic pathways from enzyme data. Biochim. Biophys. Acta Proteins Proteom..

[20] Tipton K.F. (2018). 90 years of monoamine oxidase: Some progress and some confusion. J. Neural Transm..

[21] Orsi B.A., Cleland W.W. (1972). Inhibition and kinetic mechanism of rabbit muscle glyceraldehyde-3-phosphate dehydrogenase. Biochemistry.

[22] Ganson N.J., Fromm H.J. (1985). Initial rate and isotope exchange studies of rat skeletal muscle hexokinase. J. Biol. Chem..

[23] Storer A.C., Cornish-Bowden A. (1976). Kinetics of rat liver glucokinase. Co-operative interactions with glucose at physiologically significant concentrations. Biochem. J..

[24] Wong M., Khirich G., Loria J.P. (2013). What’s in Your Buffer? Solute Altered Millisecond Motions Detected by Solution NMR. Biochemistry.

[25] García-Contreras R., Vos P., Westerhoff H.V., Boogerd F.C. (2012). Why in vivo may not equal in vitro—New effectors revealed by measurement of enzymatic activities under the same in vivo-like assay conditions. FEBS J..

[26] Glusker J.P., Scott R.A. (2011). Cation-activated enzymes. Encyclopedia of Inorganic and Bioinorganic Chemistry.

[27] Spina J., Bright H.J., Rosenbloom J. (1970). Purification and properties of l-malic enzyme from *Escherichia coli*. Biochemistry.

[28] Motherway M., Tipton K.F., McCarthy A.D., Couée I., Irwin J. (2002). Purification of glutamate dehydrogenase from liver and brain. Curr. Protoc. Protein Sci..

[29] Lund P., Wiggins D. (1987). Inhibition of carbamoyl-phosphate synthase (ammonia) by Tris and Hepes. Effect on *K*_a_ for *N*-acetylglutamate. Biochem. J..

[30] Van Eunen K., Bouwman J., Daran-Lapujade P., Postmus J., Canelas A.B., Mensonides F.I.C., Orij R., Tuzun I., van den Brink J., Smits G.J. (2010). Measuring enzyme activities under standardized in vivo-like conditions for systems biology: Standardized enzyme assays for systems biology. FEBS J..

[31] Balut C., vandeVen M., Despa S., Lambrichts I., Ameloot M., Steels P., Smets I. (2008). Measurement of cytosolic and mitochondrial pH in living cells during reversible metabolic inhibition. Kidney Int..

[32] Kresnowati M., Suarez-Mendez C., van Winden W., van Gulik W., Heijnen J. (2008). Quantitative physiological study of the fast dynamics in the intracellular pH of *Saccharomyces cerevisiae* in response to glucose and ethanol pulses. Metab. Eng..

[33] Nicholls R., Jerfy A., Roy A. (1974). The determination of the initial velocity of enzyme-catalysed reactions. Anal. Biochem..

[34] Kimmel J.R., Smith E.L. (1954). Crystalline papain. I. Preparation, specificity, and activation. J. Biol. Chem..

[35] Seydoux F., Malhotra O.P., Bernhard S.A., Stark G. (1974). Half-Site Reactivity. Crit. Rev. Biochem..

[36] Dixon M., Webb E.C., Thorne C.J.R., Tipton K.F. (1979). Enzymes.

[37] Elliott K.R.F., Tipton K.F. (1974). Kinetic studies of bovine liver carbamoyl phosphate synthetase. Biochem. J..

[38] Fersht A. (1985). Enzyme Structure and Mechanism.

[39] Eisenthal R., Danson M.J., Hough D.W. (2007). Catalytic efficiency and *k*_cat_/*K*_M_: A useful comparator?. Trends Biotechnol..

[40] Duggleby R.G. (1985). Estimation of the initial velocity of enzyme-catalysed reactions by non-linear regression analysis of progress curves. Biochem. J..

[41] Crompton I.E., Waley S.G. (1986). The determination of specificity constants in enzyme-catalysed reactions. Biochem. J..

[42] Houslay M.D., Tipton K.F. (1975). Amine competition for oxidation by rat liver mitochondrial monoamine oxidase. Biochem. Pharmacol..

[43] Sevilla C.L., Fischer E.H. (1969). Purification and properties of rat muscle glycogen phosphorylase. Biochemistry.

[44] Nimmo H., Tipton K. (1982). Fructose-bisphosphatase from ox liver. Methods Enzymology.

[45] Garfinkel L., Kohn M.C., Garfinkel D. (1979). Computer simulation of the fructose bisphosphatase/phosphofructokinase couple in rat liver. Eur. J. Biochem..

[46] McCarthy A.D., Walker J.M., Tipton K.F. (1980). Purification of glutamate dehydrogenase from ox brain and liver. Evidence that commercially available preparations of the enzyme from ox liver have suffered proteolytic cleavage. Biochem. J..

[47] Couée I., Tipton K.F. (1991). The sulphydryl groups of ox brain and liver glutamate dehydrogenase preparations and the effects of oxidation on their inhibitor sensitivities. Neurochem. Res..

[48] Johnson D.R., Lambright D.G., Wong S.S. (1985). Lactose synthase: Effect of *α*-lactalbumin on substrate activity of *N*-acylglucosamines. Biochim. Biophys. Acta Protein Struct. Mol. Enzymol..

[49] Rimmer M.A., Nadeau O.W., Artigues A., Carlson G.M. (2018). Structural characterization of the catalytic *γ* and regulatory *β* subunits of phosphorylase kinase in the context of the hexadecameric enzyme complex: Structure of *β* and *γ* Subunits of Phosphorylase Kinase. Protein Sci..

[50] Funk M.A., Judd E.T., Marsh E.N.G., Elliott S.J., Drennan C.L. (2014). Structures of benzylsuccinate synthase elucidate roles of accessory subunits in glycyl radical enzyme activation and activity. Proc. Natl. Acad. Sci. USA.

[51] Yee A., Wu L., Liu L., Kobayashi R., Xiong Y., Hall F.L. (1996). Biochemical characterization of the human cyclin-dependent protein kinase activating kinase. Identification of p35 as a novel regulatory subunit. J. Biol. Chem..

[52] Uemura S., Kihara A., Inokuchi J.I., Igarashi Y. (2003). Csg1p and newly identified Csh1p function in mannosylinositol phosphorylceramide synthesis by interacting with Csg2p. J. Biol. Chem..

[53] Wendeler M., Werth N., Maier T., Schwarzmann G., Kolter T., Schoeniger M., Hoffmann D., Lemm T., Saenger W., Sandhoff K. (2006). The enzyme-binding region of human GM2-activator protein. FEBS J..

[54] Skropeta D. (2009). The effect of individual N-glycans on enzyme activity. Bioorg. Med. Chem..

[55] Shauchuk A., Szulc B., Maszczak-Seneczko D., Wiertelak W., Skurska E., Olczak M. (2020). N-glycosylation of the human *β*1,4-galactosyltransferase 4 is crucial for its activity and Golgi localization. Glycoconj. J..

[56] Yang C., Wang Q., Ding W. (2019). Recent progress in the imaging detection of enzyme activities in vivo. RSC Adv..

[57] Speers A.E., Cravatt B.F. (2004). Profiling Enzyme Activities In Vivo Using Click Chemistry Methods. Chem. Biol..

[58] dos Santos M.M., Gombert A.K., Christensen B., Olsson L., Nielsen J. (2003). Identification of in vivo enzyme activities in the cometabolism of glucose and acetate by *Saccharomyces cerevisiae* by using ^13^C-labeled substrates. Eukaryot. Cell.

[59] Ishihama Y., Schmidt T., Rappsilber J., Mann M., Hartl F.U., Kerner M.J., Frishman D. (2008). Protein abundance profiling of the *Escherichia coli* cytosol. BMC Genomics.

[60] White C.A., Oey N., Emili A. (2009). Global Quantitative Proteomic Profiling through ^18^O-Labeling in Combination with MS/MS Spectra Analysis. J. Proteome Res..

[61] Fortelny N., Overall C.M., Pavlidis P., Freue G.V.C. (2017). Can we predict protein from mRNA levels?. Nature.

[62] Yu X., Wallstrom G., Magee D.M., Qiu J., Mendoza D.E.A., Wang J., Bian X., Graves M., LaBaer J. (2013). Quantifying antibody binding on protein microarrays using microarray nonlinear calibration. Biotechniques.

[63] Gomez N., Unzeta M., Tipton K.F., Anderson M.C., O’Carroll A.M. (1986). Determination of monoamine oxidase concentrations in rat liver by inhibitor binding. Biochem. Pharmacol..

[64] McDonald A.G., Keith K.F. (2001). Enzymes: Irreversible Inhibition. Encyclopedia of the Life Sciences (eLS).

[65] Hanson A.D., McCarty D.R., Henry C.S., Xian X., Joshi J., Patterson J.A., García-García J.D., Fleischmann S.D., Tivendale N.D., Millar A.H. (2021). The number of catalytic cycles in an enzyme’s lifetime and why it matters to metabolic engineering. Proc. Natl. Acad. Sci. USA.

[66] van Eunen K., Bakker B.M. (2014). The importance and challenges of in vivo-like enzyme kinetics. Perspect. Sci..

[67] Tipton K., Eisenthal R.A., Danson M.J. (2002). Principles of enzyme assay and kinetic studies. Enzyme Assays: A Practical Approach.

[68] Warren G.B., Tipton K.F. (1974). Pig liver pyruvate carboxylase. Purification, properties and cation specificity. Biochem. J..

[69] Warren G.B., Tipton K.F. (1974). Pig liver pyruvate carboxylase. The reaction pathway for the carboxylation of pyruvate. Biochem. J..

[70] Mitchison T.J. (2019). Colloid osmotic parameterization and measurement of subcellular crowding. Mol. Biol. Cell.

[71] Zhou G.Q., Zhong W.Z. (2005). Diffusion-Controlled Reactions of Enzymes: A Comparison between Chou’s Model and Alberty-Hammes-Eigen’s Model. Eur. J. Biochem..

[72] Fersht A. (1984). Measurement and magnitude of enzymatic rate constants. Enzyme Structure and Mechanism.

[73] Jevtic S., Anders J. (2017). A qualitative quantum rate model for hydrogen transfer in soybean lipoxygenase. J. Chem. Phys..

[74] Shahid S., Hassan M.I., Islam A., Ahmad F. (2017). Size-dependent studies of macromolecular crowding on the thermodynamic stability, structure and functional activity of proteins: In vitro and in silico approaches. Biochim. Biophys. Acta Gen. Subj..

[75] Pastor I., Pitulice L., Balcells C., Vilaseca E., Madurga S., Isvoran A., Cascante M., Mas F. (2014). Effect of crowding by Dextrans in enzymatic reactions. Biophys. Chem..

[76] Wilcox X.E., Chung C.B., Slade K.M. (2021). Macromolecular crowding effects on the kinetics of opposing reactions catalyzed by alcohol dehydrogenase. Biochem. Biophys. Rep..

[77] Balbo J., Mereghetti P., Herten D.P., Wade R. (2013). The Shape of Protein Crowders is a Major Determinant of Protein Diffusion. Biophys. J..

[78] Vogel K., Greinert T., Reichard M., Held C., Harms H., Maskow T. (2020). Thermodynamics and Kinetics of Glycolytic Reactions. Part II: Influence of Cytosolic Conditions on Thermodynamic State Variables and Kinetic Parameters. Int. J. Mol. Sci..

[79] Poggi C.G., Slade K.M. (2015). Macromolecular crowding and the steady-state kinetics of malate dehydrogenase. Biochemistry.

[80] Peters T. (1985). Serum Albumin. Advances in Protein Chemistry.

[81] Liu B., Poolman B., Boersma A.J. (2017). Ionic Strength Sensing in Living Cells. ACS Chem. Biol..

[82] Altamash T., Ahmed W., Rasool S., Biswas K.H. (2021). Intracellular Ionic Strength Sensing Using NanoLuc. Int. J. Mol. Sci..

[83] Castañeda Agulló M., del Castillo L.M., Whitaker J.R., Tappel A.L. (1961). Effect of Ionic Strength on the Kinetics of Trypsin and Alpha Chymotrypsin. J. Gen. Physiol..

[84] Nørby J.G., Esmann M. (1997). The Effect of Ionic Strength and Specific Anions on Substrate Binding and Hydrolytic Activities of Na,K-ATPase. J. Gen. Physiol..

[85] Neville W.M., Eyring H. (1972). Hydrostatic Pressure and Ionic Strength Effects on the Kinetics of Lysozyme. Proc. Natl. Acad. Sci. USA.

[86] Epstein W. (2003). The roles and regulation of potassium in bacteria. Progress in Nucleic Acid Research and Molecular Biology.

[87] Brooks S., Storey K. (1991). Where is the glycolytic complex? A critical evaluation of present data from muscle tissue. FEBS Lett..

[88] Haldane J.B.S. (1957). Graphical Methods in Enzyme Chemistry. Nature.

[89] Dowd J.E., Riggs D.S. (1965). A comparison of estimates of Michaelis-Menten kinetic constants from various linear transformations. J. Biol. Chem..

[90] De Miguel Merino F. (1974). A new method for determining the Michaelis constant. Biochem. J..

[91] Eisenthal R., Cornish-Bowden A. (1974). The direct linear plot. A new graphical procedure for estimating enzyme kinetic parameters. Biochem. J..

[92] Cornish-Bowden A., Eisenthal R. (1974). Statistical considerations in the estimation of enzyme kinetic parameters by the direct linear plot and other methods. Biochem. J..

[93] Wilkinson G. (1961). Statistical estimations in enzyme kinetics. Biochem. J..

[94] Cleland W.W. (1963). Computer Programmes for Processing Enzyme Kinetic Data. Nature.

[95] Atkins G.L., Nimmo I.A. (1975). A comparison of seven methods for fitting the Michaelis-Menten equation. Biochem. J..

[96] Cornish-Bowden A. (2001). Detection of Errors of Interpretation in Experiments in Enzyme Kinetics. Methods.

[97] Selwyn M. (1995). Michaelis-Menten data: Misleading textbook examples. Biochem. Educ..

[98] Orsi B.A., Tipton K.F. (1979). [8] Kinetic analysis of progress curves. Methods Enzymology.

[99] Goudar C.T., Sonnad J.R., Duggleby R.G. (1999). Parameter estimation using a direct solution of the integrated Michaelis-Menten equation. Biochim. Biophys. Acta.

[100] Schnell S., Mendoza C. (1997). Closed Form Solution for Time-dependent Enzyme Kinetics. J. Theoret. Biol..

[101] Zavrel M., Kochanowski K., Spiess A.C. (2010). Comparison of different approaches and computer programs for progress curve analysis of enzyme kinetics. Eng. Life Sci..

[102] Gadagkar S.R., Call G.B. (2015). Computational tools for fitting the Hill equation to dose–response curves. J. Pharmacol. Toxicol. Methods.

[103] Kirtley M.E., Koshland D.E. (1967). Models for cooperative effects in proteins containing subunits. Effects of two interacting ligands. J. Biol. Chem..

[104] Bardsley W.G., Childs R.E. (1975). Sigmoid curves, non-linear double-reciprocal plots and allosterism. Biochem. J..

[105] Solano-Muñoz F., Bardsley W.G., Indge K.J. (1981). The probability that complex enzyme kinetic curves can be caused by activators or inhibitors. Biochem. J..

[106] Storer A.C., Cornish-Bowden A. (1976). Concentration of MgATP^2-^ and other ions in solution. Calculation of the true concentrations of species present in mixtures of associating ions. Biochem. J..

[107] Cleland W. (1995). [7] Kinetic method for determination of dissociation constants nucleotide complexes. Methods Enzymology.

[108] Elliott K.R.F., Tipton K.F. (1974). A kinetic analysis of enzyme systems involving four substrates. Biochem. J..

[109] Levitzki A., Koshland D. (1976). The Role of Negative Cooperativity and Half-of-the-Sites Reactivity in Enzyme Regulation. Current Topics in Cellular Regulation.

[110] Dixon H.B.F., Tipton K.F. (1973). Negatively co-operative ligand binding. Biochem. J..

[111] Trowbridge C.G., Krehbiel A., Laskowski M. (1963). Substrate Activation of Trypsin. Biochemistry.

[112] Spears G., Sneyd J.G.T., Loten E.G. (1971). A method for deriving kinetic constants for two enzymes acting on the same substrate. Biochem. J..

[113] Holt A. (2018). On the practical aspects of characterising monoamine oxidase inhibition in vitro. J. Neural Transm..

[114] Vaux D.L. (2012). Know when your numbers are significant. Nature.

[115] Vaux D.L. (2014). Basic Statistics in Cell Biology. Annu. Rev. Cell Dev. Biol..

[116] Halling P., Fitzpatrick P.F., Raushel F.M., Rohwer J., Schnell S., Wittig U., Wohlgemuth R., Kettner C. (2018). An empirical analysis of enzyme function reporting for experimental reproducibility: Missing/incomplete information in published papers. Biophys. Chem..

